# Complications of Percutaneous Thermal Ablation of Primary and Metastatic Lung Tumors: Review of Prevention and Management Strategies

**DOI:** 10.3390/cancers18203284

**Published:** 2026-10-12

**Authors:** Guo Yuan How, Uei Pua

**Affiliations:** Department of Diagnostic Radiology, Tan Tock Seng Hospital, Singapore 308433, Singapore

**Keywords:** lung cancer, ablation, prevention, complications

## Abstract

Pulmonary malignancies encompassing both primary lung cancer and metastatic lesions are a leading cause of cancer-related mortality globally. Percutaneous thermal ablation of primary and metastatic lung tumors using radiofrequency ablation, microwave ablation, or cryoablation has emerged as an established minimally invasive treatment modality for selected patients unsuitable for surgery or requiring lung preservation. Although most lung ablation complications are self-limiting, some adverse events may result in long-term morbidity or mortality. Post-ablation pneumothorax has the highest reported incidence, with pooled incidences of 37–44%, although rates may vary according to patient cohorts and study definitions. As indications for percutaneous thermal ablation of pulmonary lesions expand to include complex lesions or higher-risk patients, the prevention and management of procedure-related complications remain central to safe clinical practice. Although prior reviews have described the techniques, oncological outcomes, and complication profiles of image-guided lung ablation, less emphasis has been placed on integrating practical complication prevention and management strategies throughout the periprocedural workflow. This narrative review aims to synthesize various complication prevention and management strategies for percutaneous thermal ablation of both primary and metastatic pulmonary lesions. The review focuses on both common and rare complications, and various aspects of complication prevention such as patient/lesion selection, procedural planning, and anesthesia/respiratory control. A comprehensive understanding of complication profiles and mitigation strategies is essential to optimize procedural safety, guide patient selection, and establish ablative techniques as a pillar in multidisciplinary thoracic oncology care.

## Simple Summary

Percutaneous thermal ablation is an established minimally invasive therapy for selected patients with primary and metastatic lung tumors who require pulmonary tissue preservation or are not suitable for surgical treatment. While the procedure is generally safe, complications such as pneumothorax, bleeding, and infection can occur. While existing reviews have described the indications, techniques, and outcomes of lung thermal ablation, fewer have emphasized practical strategies to prevent and manage complications. Our review synthesizes current evidence on periprocedural strategies to prevent complications and provides an overview of common and rare complications, measures to reduce their incidence, and evidence-based management principles. This comprehensive summary aims to improve patient selection and procedure planning, support safer clinical practice, and identify potential areas where further research can be performed in the field.

## 1. Introduction

Pulmonary malignancies, encompassing both primary lung cancer and pulmonary metastases, represent a major worldwide health challenge and remain a leading cause of cancer-related deaths globally. Primary lung malignancy was the most frequently diagnosed cancer in 2022 [1]: GLOBOCAN 2022 estimates 2.48 million new cases and 1.8 million deaths associated with primary lung cancer, double the mortality of colorectal cancer [2,3]. Non-small cell lung cancer (NSCLC) accounts for 80–85% of all primary lung malignancies, and patients demonstrate poor survival outcomes with a reported 5-year overall survival of 26.4% across all NSCLC stages [4]. The lung is the most common site of hematogenous metastasis, with autopsy studies revealing pulmonary metastasis in up to half of all cancer decedents [5]. While surgery remains the standard of care for resectable early-stage lung cancer and oligometastatic pulmonary metastasis, substantial proportions of patients are not suitable for surgical resection due to factors such as comorbidities or age. Treatment options such as SBRT and thermal ablation are crucial in managing patients unsuitable for surgery, with observational evidence demonstrating favorable outcomes with both treatment modalities [6,7].

Percutaneous image-guided thermal ablation of pulmonary lesions can be classified into radiofrequency ablation (RFA), microwave ablation (MWA), and cryoablation. RFA involves delivering an alternating current through a needle electrode to generate frictional heat within the lesion to achieve coagulative necrosis and was the first percutaneous thermal modality to gather substantial evidence for treating pulmonary lesions. However, its efficacy is limited by the heat-sink effect. MWA generates heat through electromagnetic agitation of water molecules, achieving far higher temperatures than RFA. Critically, the lack of dependence on tissue electrical conductivity renders MWA largely immune to the heat-sink effect, allowing for more predictable ablation zones and making MWA well suited for larger tumors [8]. However, general anesthesia or moderate sedation is commonly used for patients due to thermal pain. Cryoablation uses alternating freeze–thaw cycles to induce tissue necrosis, commonly using high-pressure argon expansion through the Joule–Thomson effect for freezing. Thawing may be passive or actively assisted depending on the cryoablation system, with varying numbers and durations of freeze–thaw cycles between devices and institutional protocols [9]. Key advantages of this ablative modality include real-time ice ball visualization, preservation of collagen scaffolding adjacent to the ablation zone, allowing for safer use near critical structures and the feasibility of performing the procedure under moderate sedation [9].

Although previous reviews have described the indications, technical methods, oncological outcomes and complications of image-guided ablation of pulmonary malignancies [10,11,12,13,14,15,16], fewer have integrated contemporary evidence across RFA, MWA and cryoablation within a practical periprocedural framework. As indications for image-guided thermal ablation of pulmonary lesions expand [17], a comprehensive synthesis of preventive and management strategies for lung ablation complications is important (Table 1). This article aims to provide a comprehensive review of the prevention and management of the various image-guided thermal ablative techniques in the management of primary lung cancer and pulmonary metastasis. This review is organized around a practical periprocedural approach integrating patient selection, lesion selection, procedural planning, complication-prevention strategies, and evidence-based management of both common and rare complications.

This narrative review referenced various peer-reviewed articles identified through focused PubMed searches using a combination of MeSH and Boolean methods. Search terms related to pulmonary malignancy, pulmonary metastases, thermal ablation, radiofrequency ablation, microwave ablation, cryoablation, complications, adverse events, safety, risk factors, prevention, and management were included. A representative search strategy was: (“pulmonary malignancy” OR “pulmonary malignancies” OR “pulmonary metastasis” OR “pulmonary metastases”) AND (“thermal ablation” OR “radiofrequency ablation” OR “microwave ablation” OR “cryoablation”) AND (“complications” OR “adverse events” OR “safety” OR “risk factors” OR “prevention” OR “management”). The search focused on articles published within the last 15 years and was last updated in July 2026, with selected articles referenced from earlier years. Relevant clinical practice guidelines and the reference lists of included articles were also reviewed to identify additional articles. Meta-analyses, clinical practice guidelines, systematic reviews, and larger clinical series were preferentially included where available. Smaller retrospective studies, case series, or case reports were referenced when they provided relevant information regarding rare complications or emerging preventive and management strategies when higher-level evidence was unavailable. Evidence from related transthoracic procedures was selectively considered when directly relevant evidence from image-guided lung ablation was unavailable, and it was identified as indirect evidence. No formal risk-of-bias assessment or systematic study selection was performed given the narrative nature of this review. The literature search was limited to PubMed, which may have resulted in the omission of relevant studies indexed in other databases. The risk factors, prevention, and management of these complications are summarized in Table 1.

Complication reporting after image-guided thermal ablation of lung malignancies is heterogeneous, with considerable variation in definitions of adverse events, grading systems, and follow-up durations across studies. This review references established Interventional Radiology adverse event frameworks, including the Society of Interventional Radiology (SIR) adverse event classification [18] and the modified Cardiovascular and Interventional Radiological Society of Europe (CIRSE) classification system [19]. The modified CIRSE classification system stratifies complications according to their clinical consequences, such as additional therapeutic requirements, prolonged hospitalization, permanent sequelae, or death, and classifies complications temporally (intraprocedural, periprocedural, or delayed).

Expected post-procedural findings without adverse clinical consequences should be distinguished from clinically significant complications. However, given the inter-study variation in definitions and classification systems, reported complication rates and severity grades are retained according to the definitions used in the original publications rather than retrospectively reclassified. Complication rates are reported per patient or per ablation session, where available, along with the timing of complications. Direct complication rates between studies should be interpreted with caution. Standardized use of contemporary adverse-event classification systems in studies would benefit inter-study comparison of complication severity and rates.

**Table 1:** Summary of the risk factors, prevention and management of common and rare complications of percutaneous thermal lung ablation.

Complication	Risk Factors	Prevention Strategies	Management	Modality-Specific Considerations
Pneumothorax	Older age, male sex, absence of prior lung surgery, greater number of tumors ablated, longer aerated lung tract, emphysema, reduced lung diffusion capacity, higher body mass index [20,21,22]	Careful patient selection, shortest safe needle trajectory, minimize pleural punctures, tract cauterization, gelatin sponge tract embolization, pleural blood patch [12,23,24,25]	Observation, supplemental oxygen, needle aspiration, chest tube insertion, investigate persistent air leak for bronchopleural fistula [21,26,27]	Reported with all modalities. Limited direct comparative evidence. Tract cauterization demonstrated benefit after MWA, and gelatin-sponge tract embolization following RFA.
Pulmonary hemorrhage and hemoptysis	Small lesions (<1.5 cm), basal or middle lung lesions, >2.5 cm aerated lung traversed, vessel traversal, multi-tined electrodes [28]	Careful trajectory planning, avoid pulmonary vessels, minimize lung traversal [28]	Supportive care, bleeding lung-dependent positioning, airway protection, transcatheter coil embolization for pseudoaneurysm or refractory bleeding [28,29]	Reported with all modalities. Current evidence is predominantly derived from RFA.
Pleural effusion	Cluster electrode use, tumor-to-pleura distance < 1 cm [21]	Careful procedural planning	Observation for small effusions, pleural drainage if symptomatic or large [21]	Reported following RFA and MWA. Tract cauterization may reduce pleural effusion following MWA.
Chest wall thermal injury	Pleural-abutting lesions, high thermal exposure, grounding pad-related injury (radiofrequency ablation) [30,31,32]	Appropriate grounding pad placement, maintain adequate distance from chest wall, consider cryoablation for pleural-based lesions [30,31]	Analgesia, conservative treatment, nerve block for persistent pain, supportive treatment for burns or rib fractures [32,33]	Grounding-pad burns are specific to RFA. Cryoablation may be advantageous for lesions near the pleura due to preservation of collagenous architecture.
Infection	Advanced age, recent chemotherapy [34,35]	Careful patient selection, close post-procedural surveillance [35]	Antibiotics, percutaneous or endoscopic drainage, voriconazole for invasive pulmonary aspergillosis [35]	Reported following RFA and MWA. Invasive pulmonary aspergillosis has been described following MWA.
Post-ablation syndrome	-	-	Supportive management; suspect infection if fever is persistent/worsening [36]	No established modality-specific difference.
Systemic air embolism	Bronchovascular fistula, pulmonary vein puncture, coughing during needle placement, multiple pleural passes [37]	Avoid coughing, avoid pulmonary vein traversal, minimize pleural punctures [37,38]	High-flow oxygen, appropriate patient positioning, cardiopulmonary resuscitation if required, hyperbaric oxygen therapy [37,39]	No established modality-specific difference.
Persistent air leak/alveolar-pleural or bronchopleural fistula	Pleural-abutting tumors, pre-existing lung disease, prior lung surgery, severe infection [26]	Judicious ablation near pleura, prompt treatment of infection, avoid pleural-abutting trajectories [26]	Chest drainage, pleurodesis, endobronchial valve or silicone embolus, surgery [26,27,40]	Reported across thermal ablation. Fatal BPF has been described following MWA. No established comparative difference.
Severe hemothorax and vascular injury	Pulmonary artery or intercostal artery injury, posterior lesion access [41,42]	Careful trajectory planning, avoid vascular structures, puncture near superior rib margin, monitor for new pleural fluid [41]	Transcatheter arterial or coil embolization, supportive care [42,43]	No established modality-specific difference.
Needle-tract tumor seeding	Lack of tract ablation during applicator removal [44,45]	Tract ablation during applicator removal [46]	Repeat ablation or surgical excision where appropriate [44,45]	Published evidence is largely limited to case reports following cryoablation. Tract ablation is a preventive strategy.
Retained ablation probe tip	Probe advancement through calcified tissue or cartilage, incompatible coaxial systems, difficult electrode retraction due to charring [47]	Avoid rigid tissues, ensure device compatibility, careful deployment and retraction of expandable electrodes [47]	Conservative management, percutaneous retrieval, surgical removal when appropriate [47,48]	Device-specific mechanisms include MWA antenna fracture and entrapment of expandable RFA electrode tines.

## 2. Current Status of Lung Ablation

RFA, MWA, and cryoablation differ in their tumoricidal mechanisms with resultant varying strengths and weaknesses. A systematic review performed by Jiang et al. in 2018 [49] demonstrated significantly better local tumor control in patients who underwent RFA/MWA compared to cryoablation. While patients who underwent MWA were found to have a lower proportion of local progression compared to those who underwent RFA (10.9% vs. 19.8%), no significant difference was demonstrated. Additionally, cryoablation was found to have the lowest proportion of major complications compared to RFA and MWA (4.6% vs. 11.6% and 22.5%, respectively) [49]. A recent systematic review across all three ablation modalities by Zhang et al. found that MWA demonstrated the highest local control and long-term survival, particularly for tumors larger than 3 cm [50]. RFA was found to be effective in treating small tumors (≤3 cm) [50]. In treating small tumors (≤3 cm) [50], cryoablation may be preferred for lesions near critical structures and had the lowest complication rate with no treatment-related mortality [50]. However, cross-modality outcome comparisons should be interpreted with caution, due to heterogeneity in patient cohorts, tumor characteristics, operator experience, procedural techniques, follow-up durations, and definitions of complications. Additionally, selection bias may exist in the selection of treatment modality. Comparisons of these observational studies therefore do not establish the superiority of one ablation modality over another. These modality-specific characteristics may also influence procedural safety and complication profiles, as discussed in subsequent sections.

## 3. Indications and Outcomes

### 3.1. Primary Lung Cancer

Evidence for ablation in primary lung cancer predominantly centers around early-stage NSCLC (stage 1A). Thermal ablation is recognized as a locoregional treatment option for selected patients with medically inoperable early-stage NSCLC, along with other non-surgical therapies such as stereotactic body radiotherapy (SBRT). A SEER analysis comparing ablation and SBRT in inoperable stage 1A NSCLC (6395 patients) demonstrated no significant difference in overall survival between the two treatment groups [51]. Evaluating surgery and ablation (RFA and MWA) for stage 1A NSCLC, a systematic review and meta-analysis of 8 studies (792 patients) found no significant differences in 1- to 5-year overall survival or cancer-specific survival between both treatment modalities [52]. However, surgical resection had better 1- and 2-year disease-free survival [52]. Surgery remains the preferred option in this patient population, but RFA and MWA remain justified in medically inoperable patients [52]. Cryoablation has also demonstrated promise in this setting. Rehman et al. reported a 1- and 3-year local progression-free survival of 91.8% and 89.4%, and overall survival of 100% and 94.7% in 176 medically inoperable stage 1A NSCLC patients with lesions < 2 cm [53].

### 3.2. Pulmonary Metastases

Curative intent, achieving a chemotherapy holiday in oligometastatic disease, and controlling oligoprogressive disease represent the key indications for ablation in pulmonary metastasis [54]. A systematic review and meta-analysis published by Nguyenhuy et al. reported a pooled median overall survival of 5.13 years and a 1-year local control rate of 91% [55]. RFA has shown promise as an effective technique for managing colorectal pulmonary metastasis. A systematic review of 8 studies evaluating RFA of colorectal pulmonary metastasis (903 patients) found 1-, 3-, and 5-year survival ranges of 84–95%, 35–72%, and 20–54%. Overall survival in the included patients ranged from 31 to 67 months [56]. Cryoablation was also found to be effective in treating pulmonary metastasis, achieving local tumor control of 96.6% and 94.2% at 6 and 12 months, respectively, and a 1-year overall survival of 97.5% [57]. Currently, lesion size remains a key driver of success in local tumor control following ablation of pulmonary metastasis, with local tumor control rates approaching 90% in lesions up to 3.5 cm. Overall survival greater than 5 years has been reported in selected patients following pulmonary thermal ablation. However, direct comparisons with pulmonary metastasectomy are limited by variations in patient selection and the observational nature of the evidence [58].

The main indications and clinical outcomes for thermal ablation of primary lung cancer and pulmonary metastases are summarized in Table 2. These indications provide important context for patient and lesion selection, which are closely linked to procedural safety and complication risk.

## 4. Common Complications

### 4.1. Pneumothorax

Pneumothorax is the most frequently reported event post image-guided pulmonary ablation, arising from disruption of the pleural lining and parenchymal puncture along the ablation tract. While pneumothorax is traditionally reported as the most common complication of image-guided pulmonary ablation, its classification remains nuanced. The NCCN guidelines define self-limiting pneumothorax as an expected procedural event and reserve the classification of post-ablation complications for pneumothoraces requiring further intervention. The pooled prevalence of pneumothorax was found to be up to 37% (1916 RFA sessions) in a prior systematic review and meta-analysis [20]. Similar rates of post-ablation pneumothorax were demonstrated in another systematic review and meta-analysis (44%, 24 studies), with half of the patients requiring chest tube insertion [55]. The variability in incidence of post-ablation pneumothorax and chest tube insertion likely reflects inherent heterogeneity in ablation modality, thresholds for chest drain insertion, patient and lesion characteristics, and outcome reporting methods.

Recognized risk factors for developing post-ablation pneumothorax include increased age, male gender, absence of prior pulmonary surgical resection, greater number of tumors ablated, and increased length of aerated lung traversed [20,21]. Various techniques have been described to reduce the incidence of pneumothorax after percutaneous pulmonary puncture. Dassa et al. demonstrated significantly lower rates of pneumothorax (34% vs. 62%) following gelatin sponge slurry tract embolization in patients following image-guided RFA of pulmonary lesions [25]. Tract cauterization during withdrawal of the ablation probe was found to reduce enlarging and delayed pneumothorax and pleural effusion following MWA [24]. Novel techniques have also been proposed. Preliminary data involving tract embolization by injection of a pleural blood patch through a tandem needle adjacent to the ablation applicator have shown to be a safe and effective method for avoiding chest drain insertion post-ablation [12].

Small asymptomatic pneumothoraces are usually conservatively managed with close interval monitoring and oxygen supplementation. Up to 20% of patients require aspiration or chest drain insertion [21], predominantly due to enlarging or symptomatic pneumothoraces. Persistent air leak refractory to drainage should raise concerns for an underlying bronchopleural or alveolar-pleural fistula.

### 4.2. Pulmonary Hemorrhage and Hemoptysis

Alveolar and parenchymal hemorrhage occurs due to damage to blood vessels and thermal injury to the ablation zone microvasculature. While most episodes of bleeding are self-limiting, hemorrhage can rarely be life-threatening. In a prior case series by Nour-Eldin et al. involving 248 RFA sessions, intraparenchymal hemorrhage occurred in 17.7% of patients and hemoptysis in 16.1% [28]. One patient died from massive bleeding (0.4%) [28]. Self-limiting hemoptysis may be seen in pulmonary cryoablation, with an incidence of 37–43% and grade 1 in approximately 85% of cases [59,60].

Patient selection and technique modification to avoid traversing vessels are key preventive measures for reducing intra-procedural or post-procedural pulmonary hemorrhage and hemoptysis. Risk factors include small lesions (<1.5 cm), basal and middle zone lesions, traversing >2.5 cm of lung parenchyma, traversal of pulmonary vessels, and use of multi-tined ablation probes [28].

Most patients respond to non-invasive measures such as positioning the bleeding side down, breath-holding, and supportive care. Patients with massive hemoptysis require endotracheal intubation for airway protection. Transcatheter coil embolization remains the definitive treatment in patients with pulmonary artery pseudoaneurysm [29] or refractory bleeding.

### 4.3. Pleural Effusion

Reactive pleural effusion is commonly seen after lung ablation and usually does not warrant further intervention. Post-ablation pleural effusion results from the inflammatory response to thermal injury following pleural irritation. Prior studies involving 224 RFA ablation sessions of pulmonary lesions found post-ablation pleural effusion in 19% of sessions [21], and in 2.9% of MWA sessions when pleural drainage was required [61].

Risk factors for post-ablation reactive pleural effusion include the use of a cluster electrode and a shorter distance to the nearest pleura (tumor-to-chest-wall distance < 1 cm) [21,61]. Tract cauterization was also found to reduce post-ablation pleural effusion [24]. Most reactive pleural effusions resolve spontaneously, while large or symptomatic effusions warrant pleural drainage.

### 4.4. Chest Wall Thermal Injury

Chest wall thermal injury can occur due to 2 distinct mechanisms: grounding-pad skin burns and direct thermal injury. A prior study by Huffman et al. found an incidence of 5–33% for first-degree burns and 0.1–3.2% for second- or third-degree burns, secondary to the development of high-current RF at the grounding pad site [31]. In contrast, direct thermal injury to the pleura and chest wall occurs when the ablation zone abuts the pleural surface. Prior animal models demonstrated pleural sloughing and ring-shaped thermal injury due to temperatures above 80 °C when the ablation zone involves the parietal pleural surface [30]. Delayed rib fractures were found to occur in 13.5% of patients when the ablation zone was near the chest wall [32]. Additionally, post-ablation neurological complications involving the intercostal nerves remain rare [33].

Grounding pad burns can be mitigated using correctly sited pads, monitoring impedance, and selecting pads with adequate surface area. Direct chest wall injury can be minimized by maintaining sufficient distance from the parietal pleura. Employment of cryoablation can be considered for lesions near the pleural lining due to preservation of the collagen scaffold, as opposed to heat-based modalities [8].

Superficial burns can be managed conservatively. Intercostal neuralgia and chest wall pain can be treated with analgesia, with consideration for nerve blocks in patients with persistent pain. Post-ablation rib fractures are usually asymptomatic and can be managed conservatively.

### 4.5. Post-Ablation Syndrome

Post-ablation syndrome is an expected inflammatory response to tissue necrosis and is largely self-limiting [36]. Patients may present with symptoms such as low-grade fever, malaise, nausea, and other flu-like symptoms post-thermal ablation. Symptoms usually manifest within the first few days and improve with supportive management. It is crucial to distinguish an expected post-ablation reaction from infection, especially in patients with clinical features indicative of infection or persistent/worsening fever.

### 4.6. Infection

Post-ablation infection ranges from pneumonia to abscess formation secondary to necrosis. A prior retrospective series by Okuma et al. found that approximately 3% of patients who underwent lung ablation with RFA developing post-ablation fever and abscesses, respectively [34]. Similar findings were demonstrated in patients after pulmonary MWA, with prevalences of post-ablation pneumonia and pulmonary abscesses of 2.9% and 0.5%, respectively [61]. Invasive pulmonary aspergillosis (IPA) represents a distinct and potentially life-threatening spectrum of post-ablation infection. A multicenter study by Huang et al. found IPA in 1.44% of patients after pulmonary MWA, with a mortality of 26.1% [35]. Careful patient selection remains central to the prevention of severe infective complications post-lung ablation. The elderly and patients with recent chemotherapy are predisposed to IPA [35]. Bacterial abscesses can be treated with targeted antibiotics and percutaneous or endoscopic drainage. IPA requires early diagnosis and prompt initiation of voriconazole as first-line therapy.

Post-ablation cavitation represents a distinct disease process from infection, although early cavitation may predispose to secondary infection. Early enlarging cavitation was found to occur in 6.3% of post-lung ablation using RFA, predisposing to secondary infection [62]. Interval CT scans to monitor post-ablation necrosis should be performed if clinically indicated. The risk of cavitation increases in lesions ≥ 20 mm from the pleura, vessel contact ≥ 3 mm, bronchus ≥ 2 mm encompassed in the ablation zone, and multitined expandable electrode ≥ 3 cm. Fever ≥ 38.5 °C, raised white blood cell count one day post-ablation, and steroid therapy were independent post-procedural factors of occurrence [62].

## 5. Rare Complications

Literature describing rare complications of lung ablation is dominated by case reports and small case series. Prior studies have attempted to classify these rare complications [13]. The majority of these rare complications can be managed conservatively, by percutaneous or endoscopic techniques, or by surgical repair.

### 5.1. Systemic Arterial Air Embolism

Systemic arterial air embolism is a rare and potentially life-threatening complication secondary to transient bronchovascular fistula or direct puncture of a pulmonary vein with resultant entry of air into the systemic arterial circulation [37]. This should be distinguished from venous air embolism, in which air enters the systemic venous circulation and predominantly affects the right heart and pulmonary circulation. The presence of air in the left heart, coronary arteries, pulmonary veins, aorta, or cerebral vessels may precipitate ventricular fibrillation, myocardial infarction, or cerebral infarction [38].

Preventive measures for systemic air embolism include avoiding coughing during needle placement, avoiding trajectories that traverse aerated lung near the pulmonary veins, and minimizing the number of pleural passes. High intraprocedural vigilance is required for early detection of systemic air embolism [39].

Upon discovery of systemic arterial air embolism, immediate cessation of the ablation and administration of high-flow oxygen should be performed. Cardiopulmonary stabilization and cardiac and neurological assessment should be performed. Prompt initiation of cardiopulmonary resuscitation may be required [37]. Patient positioning should be tailored according to the location of the air embolus and clinical circumstance. Hyperbaric oxygen therapy can also be considered for coronary or cerebral air embolism [38].

### 5.2. Persistent Air Leak (PAL), Bronchopleural Fistula (BPF) and Alveolar-Pleural Fistula (APF)

PAL may occur post lung ablation due to communication between the pulmonary airways and pleural space and is generally defined as an air leak persisting beyond 5–7 days [63,64]. BPF represents a persistent communication between the larger airways and the pleural space, while an APF represents a communication between the alveoli and pleural space.

While the current literature describes the expected site of BPF at the location of thermal ablation, it may rarely occur remotely in regions not in the proximity of the ablation zone [65]. While the incidence is rare, the condition may be life-threatening. Zheng et al. reported a post-lung MWA BPF incidence of 0.3% (2 cases of 682 MWA sessions); both patients died from the complication [26]. Proposed risk factors for developing BPF include close abutment of the tumor to the pleural lining, pre-existing pulmonary diseases, prior pulmonary surgery, and severe infection [26].

PAL post-lung ablation should prompt evaluation for BPF or APF [27]. Preventive measures include judicious ablation near the pleura, aggressive treatment of post-ablation infection, and avoiding pleural-abutting trajectories [26].

Treatment individualized according to the severity and anatomical source of the air leak is required. Initial management generally involves pleural drain insertion and treatment of infection if present. Persistent leaks may warrant pleurodesis or bronchoscopic therapy. Surgical repair may be considered in selected patients with refractory fistulae. The majority of cases resolved with insertion of chest drains, pleurodesis, endoscopic therapy, or surgery [26]. Novel techniques involving endobronchial placement of endobronchial valves have proven effective in resolving persistent pneumothorax in a series [27]. The use of endoscopic bronchial occlusion with a silicone embolus has shown promise in a patient with persistent air leak after lung RFA following chest drain placement for a month [40]. However, these innovative techniques remain confined to case reports or small case series and have not been demonstrated in larger patient cohorts.

### 5.3. Severe Hemothorax and Vascular Injury

Life-threatening bleeding can occur predominantly through two vascular mechanisms: pulmonary artery pseudoaneurysm and intercostal artery injury. Pulmonary artery pseudoaneurysms can form when the ablation is performed adjacent to a pulmonary artery branch. Pulmonary artery pseudoaneurysm is rare (reported incidence of 0.2%) and may present with delayed hemoptysis or rupture with catastrophic hemorrhage [42]. Similarly, intercostal artery injury during transthoracic puncture resulting in massive uncontrolled hemothorax remains rare but is associated with high morbidity and mortality [41].

Pulmonary artery pseudoaneurysm risk can be reduced by identifying and avoiding trajectories that traverse the vessel [42]. Intercostal artery injury risk can be minimized by avoiding posterior pulmonary lesions where the artery courses away from the rib groove and positioning the puncture as close to the superior margin of the rib as possible. Additionally, constant reassessment of the density of any new pleural fluid intraprocedurally can allow for early detection of potential hemothorax [41].

Management of pulmonary artery pseudoaneurysm requires definitive treatment with transcatheter coil embolization [29]. Active intercostal artery hemorrhage can be treated with transcatheter arterial embolization. A prior multi-institutional study achieved an 87% technical success rate. However, the 30-day mortality remains high (23%) in patients presenting with massive hemorrhage, highlighting the importance of early recognition and treatment [43].

### 5.4. Neural Injury

Thoracic nerve injury following thermal ablation of pulmonary lesions is rare but potentially severe, occurring in approximately 1–2% of thoracic ablations [33]. Phrenic nerve injury has been reported at similar rates following pulmonary RFA and MWA, at 1.3% each [66,67]. Brachial plexus and stellate ganglion (Horner syndrome) injuries are almost exclusively seen in ablation of apical lung tumors [68,69], whereas phrenic nerve injury may occur during ablation of lesions near the diaphragm [70]. As these neural structures are generally not directly visualized on CT, prevention relies on knowledge of their expected anatomical course [33]. Protective measures such as instillation of fluid or air, balloon interposition, probe manipulation, and supplementary neuromonitoring can be performed [33,71,72].

### 5.5. Diaphragmatic Injury and Diaphragmatic Hernia

Thermal injury to the diaphragm during ablation of basal or lower lobe pulmonary lesions risks perforation and delayed diaphragmatic hernia. A prior study by Alberti et al. found diaphragmatic hernia following 2.3% of lower-lobe RFA procedures at a mean of 7.8 months [73]. Risk was associated with central electrode position and placement of at least one electrode in contact with or penetrating the diaphragm [73]. Preventive measures include avoiding direct electrode contact with the diaphragm and using protective separation techniques for peridiaphragmatic lesions.

### 5.6. Interstitial Pneumonia, Acute Lung Injury, Acute Respiratory Distress Syndrome (ARDS)

Besides exacerbation of pre-existing ILD, pulmonary thermal ablation can rarely trigger de novo lung injury. Such cases are predominantly limited to isolated cases. The spectrum ranges from bronchiolitis obliterans-like reactive pneumonitis [74], interstitial pneumonia, aseptic pleuritis [75] to fulminant ARDS or massive necrosis [76,77]. Despite the low incidence, interstitial pneumonia has contributed to procedure-related mortality in large RFA cohorts (3 of 4 deaths per 1000 sessions) [78]. Older age, larger tumor size (≥2 cm), higher number of punctures, previous systemic chemotherapy, and prior external-beam radiotherapy are risk factors for severe post-ablation inflammation [75,78]. Prevention centers on careful patient selection and adjusting for modifiable risk factors.

### 5.7. Needle-Tract Tumor Seeding

Seeding of tumor along the ablation needle tract is a rare complication with evidence largely confined to case reports following cryoablation [44]. Lack of cauterization of the electrode tract may be a contributing factor [45]. Tumor implantation occurs during needle withdrawal, with patients presenting months after lung ablation. Prevention centers on tract ablation during applicator withdrawal [46]. Repeat ablation or surgery can be considered in patients with needle-tract tumor seeding.

### 5.8. Retained Ablation Probe Tip

Retention of a fractured ablation applicator tip within the lung parenchyma is one of the rarest device-related complications of image-guided ablation. Supporting literature is limited to isolated case reports, with no dedicated study to establish incidence [47]. The clinical impact on patients varies, ranging from asymptomatic incidental presentations to refractory BPF with recurrent tension pneumothoraces [48].

Preventive measures are aimed at targeting failure mechanisms directly. MWA antennas should not be advanced through rigid tissue like calcified pleural plaques or costal cartilage. Coaxial-needle compatibility should be checked prior to insertion given the fragility of the antenna and risk of fracture against rigid structures. When deploying expandable RFA electrodes, the tines should be retracted carefully and monitored for entrapment of the hooks in the shaft, with subsequent applicator fracture, which may occur due to charring.

Management of retained ablation probes is individualized with no consensus guidelines. Conservative management can be considered for asymptomatic, deeply seated, or inaccessible fragments in medically inoperable patients. Percutaneous retrieval of foreign bodies in non-vascular structures using a large-bore biopsy or trocar system under image guidance has been described [79]. Surgical removal remains the mainstay for retained foreign bodies when feasible [48].

## 6. General Principles for Preventing Complications

### 6.1. Patient Selection

Reserving image-guided ablation for appropriately selected patients is key to optimizing outcomes and reducing risks. Medically inoperable patients due to poor pulmonary function or volume, pre-existing comorbidities, or favorably located pulmonary lesions are established indications for lung ablation. Thermal ablation remains relatively low risk for lesions < 3 cm, located peripherally, and not involving major airways or vessels [54]. A greater number of punctures, reduced lung diffusion capacity, presence of emphysema, and raised BMI have been identified as risk factors for developing pneumothorax post-lung MWA [22]. The presence of emphysema and chronic obstructive pulmonary disease (COPD) predisposes patients to pneumothorax and prolonged air leak post-ablation, with emphysema being associated with chest tube placement post-MWA [61]. Patients with coexisting interstitial lung disease (ILD) developed a higher rate of pneumothorax (60.0% vs. 38.9%) and a significantly higher rate of pneumothorax requiring chest drain insertion (36.0% vs. 11.2%) compared to non-ILD patients [80]. Importantly, acute exacerbation of pre-existing ILD is a rare but potentially fatal complication post lung ablation. Ablation-related mortality of 7.1% has been seen among patients with pre-existing ILD, mostly related to acute exacerbation [80].

### 6.2. Perioperative Pharmacological Considerations

Image-guided ablation is a percutaneous transpulmonary procedure with a moderate-to-high bleeding risk. Strict adherence to societal and institutional guidelines is required, including assessment of pre-procedural laboratory parameters (platelet count and coagulation profile) and individualized management of antiplatelet and anticoagulant therapy. The Society of Interventional Radiology (SIR) and Cardiovascular and Interventional Radiological Society of Europe (CIRSE) have published consensus guidelines regarding the periprocedural management of antithrombotic therapy [81,82]. The risk of bleeding from the puncture must be weighed against the patient’s thrombotic risk [83], and this is further complicated in the oncology cohort since malignancy itself raises the risk of both thrombosis and hemorrhage [84]. Currently, no consensus exists for routine administration of antibiotic prophylaxis in patients undergoing thermal ablation of pulmonary lesions.

### 6.3. Anesthesia and Respiratory Control

Intra-procedural analgesia and sedation are crucial due to substantial procedural pain secondary to thermal stimulation of the parietal pleura [85]. Percutaneous thermal ablation of pulmonary lesions may be performed under local anesthesia with moderate sedation or under general anesthesia [86]. Pulmonary-specific nerve root blocks have also demonstrated promise in pain control during lung ablation [87]. The choice of anesthetic technique depends on patient factors, ablation modality, lesion characteristics, procedural complexity, institutional practice, and local expertise. Adequate analgesia and sedation improve patient comfort and may reduce coughing and unexpected respiratory motion during applicator placement and ablation. However, deeper levels of sedation may impair spontaneous ventilation and airway protective reflexes, necessitating appropriate airway and cardiorespiratory monitoring.

General anesthesia provides secure airway control and facilitates controlled ventilation and reproducible respiratory maneuvers such as breath-holding, which may improve the consistency and accuracy of applicator placement, particularly for small or technically challenging lesions. However, it entails additional anesthetic and ventilation-related risks. The anesthetic and respiratory-control strategy should therefore be individualized according to patient and procedural requirements rather than routinely applying general anesthesia to all patients.

### 6.4. Lesion Selection

Tumor size and location strongly influence treatment outcomes and safety. Close proximity to the pleura, pulmonary vessels and airways increases the risk of bleeding, pleural effusion and cavitation [28,62]. Larger lesions (≥3 cm) are associated with poorer local control. The chosen ablative modality should also be tailored to suit the lesion. RFA may be considered for small peripheral lesions, MWA for larger lesions, and cryoablation for tumors close to critical structures or very small lesions requiring parenchymal preservation or real-time margin monitoring [50].

### 6.5. Procedural Planning

Applicator trajectory is a vital modifiable determinant of risk. In a multi-center study involving 416 ablations, steeper pleural puncture angles were independently associated with fewer major adverse events in centrally located tumors [23]. For non-central lesions, 3 or more pleural punctures or the use of MWA were independently associated with more major adverse events [23]. Reducing the number of pleural passes, deploying ablation tract management techniques (tract cauterization, tract embolization, or pleural blood patch), and aiming for the shortest safe aerated lung trajectory have been shown to reduce pneumothorax and pleural complications [12,24,88].

Protective displacement techniques may allow for safe margins when ablating lesions adjacent to critical structures. Creation of artificial pneumothorax, balloon interposition, hydrodissection, or probe torquing not only separates the target lung from vulnerable structures, but may also provide additional thermal insulation [89]. Extrapleural air injection has been described to protect the intercostal nerves during ablation of peripheral lesions [72].

## 7. Conclusions and Future Directions

Image-guided thermal ablation is a safe and effective lung-preserving treatment technique for selected patients with primary or metastatic pulmonary lesions. Procedural complications can be reduced through careful patient selection, lesion selection, and procedural planning. Most post-ablation complications are predominantly confined to minor self-limiting events without long-term sequelae. While prior reviews have comprehensively described the indications, techniques, oncological outcomes, and complications of image-guided ablation of pulmonary malignancies, this review emphasizes practical strategies for preventing, recognizing, and managing complications throughout the patient’s journey.

Several priorities emerge. Firstly, further prospective comparative studies are required for tract-management techniques to reduce pleural complications. Pleural complications remain the most common adverse events post-lung ablation. While several tract-management techniques have demonstrated promise in reducing pleural complications, comparative evidence remains limited. Next, emerging technologies used in treatment planning and device development are likely to reshape the practice and complication profile of lung ablation. Constant updates to the literature and more studies will have to be performed in this area. As indications for lung ablation continue to broaden and minimally invasive tumoricidal techniques are increasingly incorporated into multidisciplinary pulmonary oncological care, a rigorous prevention-oriented understanding of complications will remain central to safe lung ablation.

## Figures and Tables

**Table 2:** Summary of the indications and outcomes of thermal ablation for primary lung cancer and pulmonary metastases.

Disease	Main Indications	Clinical Outcomes
Early-stage NSCLC	Medically inoperable patients; alternative to surgery or stereotactic body radiotherapy	Comparable overall survival to stereotactic body radiotherapy in medically inoperable patients; comparative evidence remains limited. Surgery remains the preferred treatment for operable patients.
Pulmonary metastases	Oligometastatic disease, oligoprogressive disease, chemotherapy holiday	High local control and favorable long-term survival in appropriately selected patients.

## Data Availability

No new data were created or analyzed in this study. Data sharing is not applicable to this article.

## Abbreviations

The following abbreviations are used in this manuscript:

PAL	Persistent air leak
BPF	Bronchopleural fistula
APF	Alveolar-pleural fistula
CT	Computed tomography
GLOBOCAN	Global Cancer Observatory
IPA	Invasive pulmonary aspergillosis
MWA	Microwave ablation
NCCN	National Comprehensive Cancer Network
NSCLC	Non-small cell lung cancer
RFA	Radiofrequency ablation
SBRT	Stereotactic body radiotherapy
SEER	Surveillance, Epidemiology, and End Results
